# Phylogeography and Domestication of Chinese Swamp Buffalo

**DOI:** 10.1371/journal.pone.0056552

**Published:** 2013-02-20

**Authors:** Xiang-Peng Yue, Ran Li, Wen-Mei Xie, Ping Xu, Ti-Cheng Chang, Li Liu, Feng Cheng, Run-Feng Zhang, Xian-Yong Lan, Hong Chen, Chu-Zhao Lei

**Affiliations:** 1 Shaanxi Key Laboratory of Molecular Biology for Agriculture, College of Animal Science and Technology, Northwest A & F University, Yangling, Shaanxi, China; 2 Department of Basic Medicine, Pingliang Medical College, Pingliang, Gansu, China; 3 Department of Dairy and Animal Science, The Center for Reproductive Biology and Health, College of Agricultural Sciences, The Pennsylvania State University, University Park, Pennsylvania, United States of America; 4 Department of East Asian Languages and Cultures, Stanford University, Stanford, California, United States of America; 5 Animal Science Department, Xinyang Agricultural College, Xinyang, Henan, China; 6 College of Life Science, Hubei Normal University, Huangshi, Hubei, China; University of Perugia, Italy

## Abstract

To further probe into whether swamp buffaloes were domesticated once or multiple times in China, this survey examined the mitochondrial DNA (mtDNA) Control Region (D-loop) diversity of 471 individuals representing 22 populations of 455 Chinese swamp buffaloes and 16 river buffaloes. Phylogenetic analysis revealed that Chinese swamp buffaloes could be divided into two distinct lineages, A and B, which were defined previously. Of the two lineages, lineage A was predominant across all populations. For predominant lineage A, Southwestern buffalo populations possess the highest genetic diversity among the three hypothesized domestication centers (Southeastern, Central, and Southwestern China), suggesting Southwestern China as the most likely location for the domestication of lineage A. However, a complex pattern of diversity is detected for the lineage B, preventing the unambiguous pinpointing of the exact place of domestication center and suggesting the presence of a long-term, strong gene flow among swamp buffalo populations caused by extensive migrations of buffaloes and frequent human movements along the Yangtze River throughout history. Our current study suggests that Southwestern China is the most likely domestication center for lineage A, and may have been a primary center of swamp buffalo domestication. More archaeological and genetic evidence is needed to show the process of domestication.

## Introduction

Water buffaloes are widespread throughout central and southern China, with the total number of buffaloes in China in 2003 reaching almost 23 million. It is the third largest population of buffalo in the world, representing 17.37% of the total bovine populations in China [1]. Domestic water buffaloes are employed as draught animals, especially in the rice planting regions of Southern China, and they also have a tremendous economic importance due to an increasing demand for their milk, meat, horns, and skin in recent years [2]. The domestic water buffalo in Asia is generally classified into two major subspecies based on body size, outward appearance, biological characteristics, and chromosome karyotype: the river buffalo (2n = 50) and the swamp buffalo (2n = 48) [3]. Previous studies on the water buffalo mtDNA Control Region (D-loop) sequence analysis showed obvious genetic differentiation between the swamp and the river buffalo [4]–[14]. It was proposed that the river buffalo was first domesticated in the western part of South Asia [3], [11], [12], while the swamp buffalo was likely domesticated in Southeastern Asia [15] and China [14], [16]. Based on *Cytb* gene sequences of the mtDNA, the number of transitions in swamp buffaloes was higher than that in river buffaloes (average number: 7.8 vs 2.4) [17]. A large variety of genetic resources of swamp buffalo, especially in Southwestern, Southeastern and Central China along the Yangtze River, have been described in terms of adaptation to environment and distribution [18]. At present, the Chinese swamp buffaloes have been divided into 14 local types and many populations based mainly on regional distribution [18]. Though there are many indigenous wild swamp buffalo populations in widespread area of China in prehistoric times [19]–[21], studies on genomics and domestication of Chinese swamp buffalo were limited [13], [14], [22]–[25]. Two mtDNA lineages A and B have been determined in the Chinese, Brazilian/Italian and South-East Asian/Australian swamp buffalo [14]. However, due to a restricted sampling from Central and Southern China, the patterns of mtDNA sequence diversity and geographical partitioning of lineage A and lineage B have not been fully resolved. In order to unveil the domestication of Chinese swamp buffalo, a deep phylogeographic analysis with more individuals and populations is required. The current study obtained swamp buffalo mtDNA Control Region sequences from 15 provinces across China, which allow us to systematically investigate the phylogeographic structure of Chinese swamp buffaloes.

## Materials and Methods

### Specimen Collection and Buffaloes Control Region Sequences Mining

**Figure 1:**
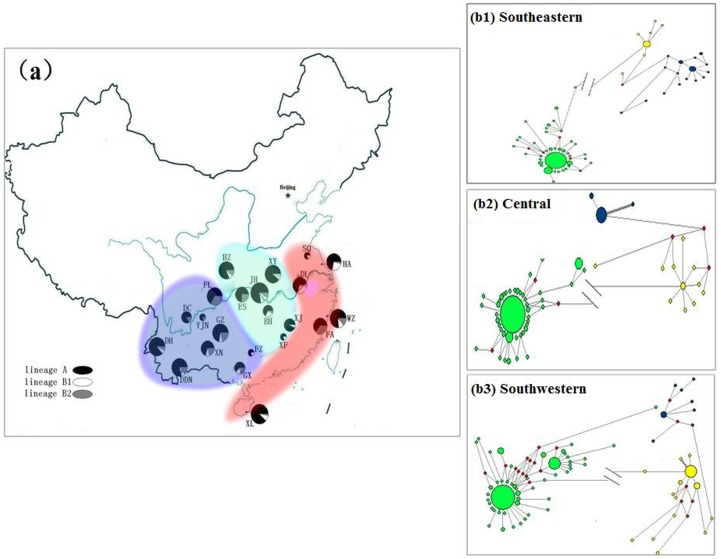
Geographic distribution and lineage composition of 22 Chinese swamp buffalo populations. (a). The area of the circle is proportional to the sample size. And the median-joining networks for three different population group defined by Fig. 1a (b).

A total of 239 fresh blood samples in this study were collected from different villages covering the entire native tract of each population and the farmers were interviewed in detail to ensure unrelatedness among the sampled individuals. We also wrote a script to retrieve all the swamp buffalo sequences deposited in NCBI database, including 119 sequences of Chinese swamp buffaloes from our previous publication (GenBank Accession Nos. DQ364160-DQ364189 and DQ658051-DQ658139) [14], and 113 D-loop sequences from different geographic regions in China available from GenBank with Accession Nos: AY702618, EF597573-EF597662 and EF053531-EF053552 (Table S1), which are in total 471 sequences of mtDNA Control Region (915 bp), representing 22 local swamp buffalo populations from 15 provinces in China (Fig. 1a, Table S1). We also obtained 5 swamp buffalo mtDNA Control Region sequences from Thailand with 853 bp long (DQ995708-DQ995712), 10 from Italy and Brazil (AY195596-AY195599, AF197218-AF197223) with 924–962 bp long, 8 from Philippines (FJ873676-FJ873683) with 494–497 bp long (Table S1).

### Amplification and Sequencing

DNA was isolated from fresh blood using a standard extraction method of phenol/chloroform [26]. The leucocyte pellets were prepared from the whole blood samples and subjected to cell lysis in a solution containing SDS and proteinase K. Protein precipitation was performed with phenol/chloroform, and genomic DNA was precipitated with isopropanol and ethanol. 915 bp mtDNA Control region sequences of swamp buffaloes were amplified with primers CB1∶5′-TAGTGCTAATACCAACGGCC-3′ and CB2∶5′-AGGCATTTTCAGTGCCTTGC-3′ [10]. A PCR reaction was performed in 50 µl reaction volume with 20 ng genomic DNA, 10 pM each primer, 0.25 mM dNTPs, 1×PCR buffer with 2.5 mM MgCl_2_ and 2.0 U *Taq* DNA polymerase (MBI). A Progene thermal cycler (PE 9600) was used for PCR amplification with the following conditions: initial denaturation at 94°C for 30 sec, followed by 35 cycles at 94°C for 1 min, 56.5°C for 1 min, and 72°C for 1 min and the final extension at 72°C for 4 min. The amplified products were purified using Wizard PCR Preps DNA purification kit (Promega) according to the manufacturer’s instructions. PCR products were sequenced using four internal primers: CB3: (5′- CCA TCA ACA CAC CTG ACC -3′), CB4: (5′- GCG AGG ACG GAT TTG ACT -3′), CB5: (5′- CAT AAC ATT AAT GTA ATA AGG GC -3′) and CB6: (5′- CCA TTC GGA GTA GTA GGG TC -3′). The sequencing reactions were carried out on an ABI 3730 automated sequencer. All new sequences produced in this study were deposited in the GenBank (accession nos GQ260217–260455).

### Data Analysis

All the mtDNA Control Region sequences were edited using the DNASTAR 5.0 package (DNASTAR, Madison, WI). The ClustalX package [27] was used for multiple alignments. All mtDNA insertions/deletions in the alignment were excluded from the analyses. Identical sequences were considered as the same haplotype. Haplotype diversity (h), nucleotide diversity (π), the F*st* and AMOVA were estimated using the Arlequin 3.5 package [28]. To demonstrate the phylogenetic clusters, the MEGA5.0 [29] was applied to construct Maximum likelihood (ML) trees, using Hasegawa-Kishino-Yano model with additional parameter of 1000 bootstrapping replicates, Gamma distribution (+G) with 8 rate categories, and evolutionarily invariable (+I).

In the ML trees, reliability percentages (RP) above 50% can be considered as strong supported. The Bayesian phylogenetic tree was also constructed using TOPALi 2.5 [30]. A complete mtDNA sequence (GenBank accession no: NC_006853) of cattle (*Bos taurus*) was used as an out-group in the phylogenetic tree construction. Then, median-joining network (MJ) was generated using the NETWORK 4.1 program [31]. All the gaps were excluded in constructing median-joining networks and parameters were set to a weight of two and threshold value of one. The network calculations were run with different values for ε [31], however the best results were obtained with ε = 0. Frequencies of haplotypes were converted into proportional areas in the figures. The pairwise mismatch distribution between buffalo sequences were generated using Dnasp 5.0 program and the pairwise F*_ST_* values were displayed by multidimensional scaling (MDS) using SPSS11.0 [12]. Divergence time and most recent common ancestor were calculated using MEGA 5.0. The mutation rate was assumed to be 32%/million years [32].

## Results

### Phylogenetic Tree Construction and Haplotype Diversity of Chinese Buffaloes

**Figure 2:**
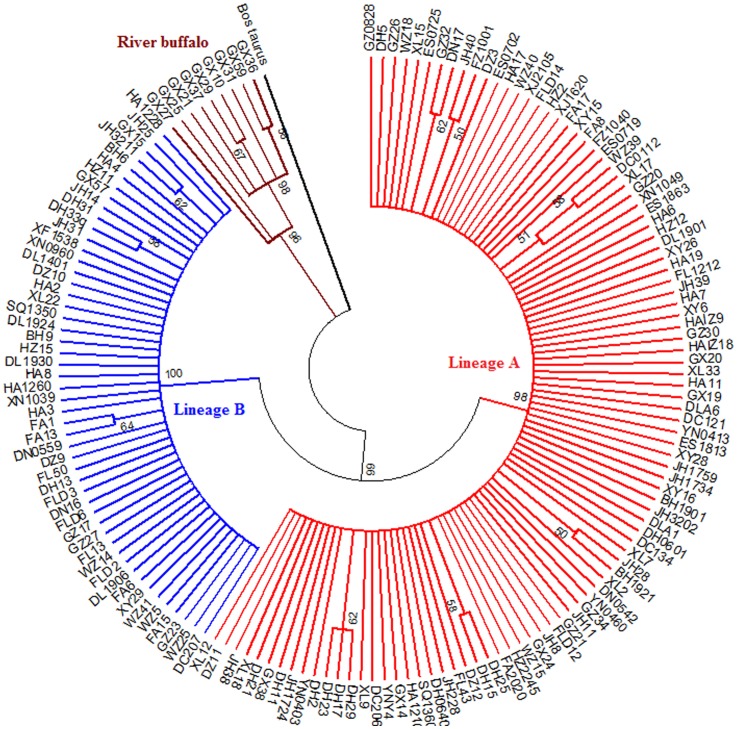
ML tree of 157 Chinese buffalo haplotype.

**Figure 3:**
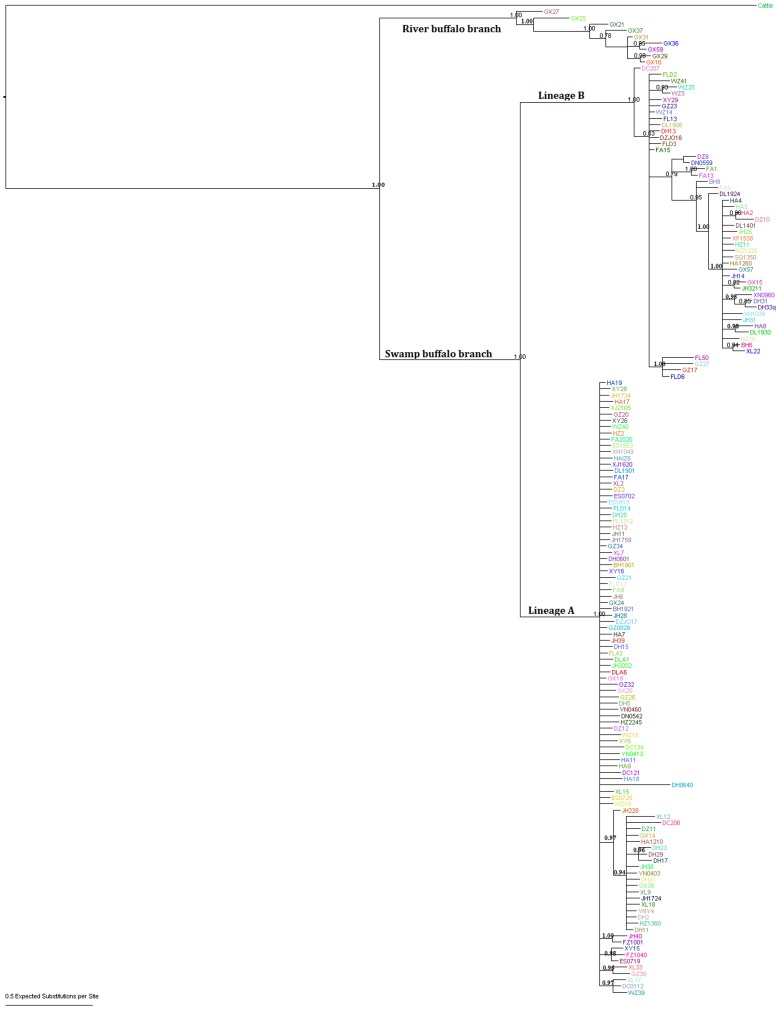
Bayesian tree based on 157 haplotypes this study.

The comparison of all 471 mtDNA Control Region sequences (915 bp) from 22 Chinese buffalo populations showed 157 haplotypes. The ML tree and Bayesian tree constructed with the 157 haplotypes clearly showed two main branches of swamp and river buffalo, respectively (Fig. 2, Fig. 3). The river buffalo branch included 9 haplotypes, representing 16 individuals from the Guangxi population, indicating the introgression of the river buffalo into the swamp buffalo due to importing the river buffalo to improve the dairy production of local swamp buffaloes. Whereas 148 swamp buffalo haplotypes can be grouped into two distinct mtDNA lineages A and B defined by Lei et al. [13], [14]. The comparison of 148 haplotypes representing 454 swamp buffalo sequences revealed 114 polymorphic nucleotide sites, including 99 transitions, 10 transversions and 5 coexistences of transition and transversion (Table S2). It is much higher than the 64 sites in the research by Lei et al. [14]. The 9 river buffalo haplotypes indicated 31 polymorphic nucleotide sites with 30 transitions and 1 transversion (data not shown). The ratios of transitions versus transversions in both swamp and river types were high, revealing a strong transition bias in buffaloes. For the swamp buffalo branch, five haplotypes were found to be the main haplotypes present in more than 10 samples. The predominant haplotype HA9 (lineage A) was observed 164 times with the highest frequency (36.04%, 164/455). The haplotypes GZ34, DH11 (lineage A), FA15 and HA4 (lineage B) were observed 22, 21, 31 and 13 times, respectively. Of the total 148 swamp buffalo haplotypes detected, 98 fall within the lineage A (352 sequences) and 50 are encountered within lineage B (103 sequences) (Table S3). Among above 148 haplotypes, 108 haplotypes are unique, only observed once. Meanwhile, 66 unique haplotypes (66/98 = 67.34%) belong to lineage A, and 42 unique (42/50 = 84.00%) haplotypes belong to lineage B. In contrast, 5 out of 9 (5/9 = 55.56%) haplotypes in river lineages occurred only once. The estimated divergence time of river and swamp type was 34998 years ago. In the swamp type, two separate maternal lineages A and B have been identified with an estimated divergence time of 17924 years.

**Figure 4:**
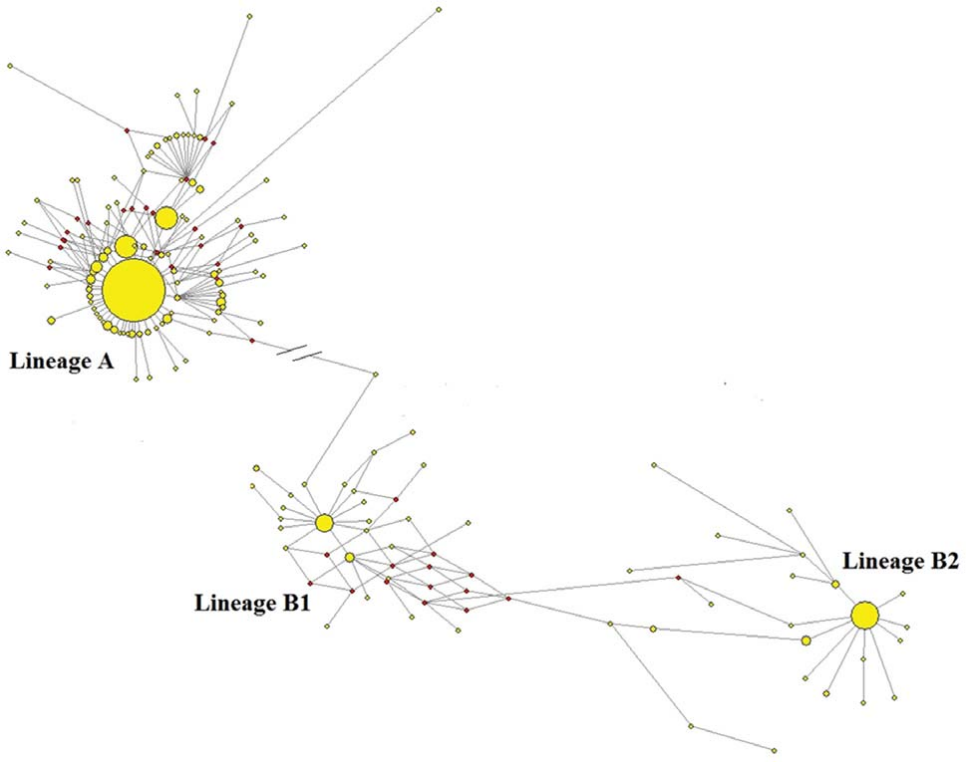
MJ network of Chinese 454 swamp buffaloes. The area of the circle is proportional to sample sizes.

In order to focus on the phylogenetics among Chinese swamp buffaloes, the median-joining network of 455 Chinese swamp buffalo samples was constructed after removing the 16 river buffaloes from the GX population (Fig. 4). As expected, the network clearly identified lineages A and B which were separated by 26 mutation steps. Interestingly, two small lineages with haplotype DH11 representing 21 individuals and GZ34 representing 22 individuals can be found in lineage A, and there are 6 and 2 mutation steps connecting with the main haplotype HA9 (164 individuals, 46.59% of the total of lineage A), respectively. Lineage A showed an obvious star-like phylogeny and was detected in 22 Chinese swamp populations (Table 1, Fig. 4). However, lineage B comprised two sublineages in the network, named B1 and B2, which are not supported in the ML tree, because of low bootstrap value (Fig. S1). The sublineages B1 and B2 also showed a star-like phylogeny with centers being HA4 (representing 13 individuals) and FA15 (representing 31 individuals), respectively. There are 20 steps between the two centers. The sublineage B1 (representing 43 individuals, 41.75% of the total of lineage B) and B2 (60 individuals, 58.25% of the total of lineage B) included 27 and 23 haplotypes, respectively (Fig. 3, Table S3). Overall, the mean number of haplotype diversity for the lineage B (0.8932±0.0253) is higher than that for the lineage A (0.7752±0.0239).

**Table 1:** Source and genetic diversity index of 455 Chinese swamp buffalo samples.

Population	Code	Geographic distribution	N	K	Lineageobserved	Haplotype diversity(SE)	Nucleotide diversity(SE)
Haizi	HA	Southeastern region (SE), Jiangsu	25	17	A, B1, B2	0.8800±0.0637	0.0183±0.0094
Shanqu	SQ	Southeastern region (SE), Jiangsu	5	5	A, B1	1.0000±0.1265	0.0157±0.0099
Dongliu	DL	Southeastern region (SE), Anhui	23	14	A, B1	0.9012±0.0512	0.0189±0.0097
Wenzhou	WZ	Southeastern region (SE), Zhejiang	30	14	A,B1, B2	0.8345±0.0638	0.0159±0.0081
Xinglong	XL	Southeastern region (SE), Jiangxi	35	19	A, B1, B2	0.8420±0.0614	0.0102±0.0053
Fu’an	FA	Southeastern region (SE), Fujian	22	9	A, B2	0.8268±0.0611	0.0202±0.0104
Binhu	BH	Central region (CR), Hunan	12	9	A, B1, B2	0.9091±0.0795	0.0204±0.0109
Jianghan	JH	Central region (CR), Hubei	36	25	A, B1, B2	0.9349±0.0327	0.0162±0.0083
Enshi	ES	Central region (CR), Hubei	20	9	A, B2	0.8579±0.0537	0.0163±0.0085
Xiajiang	XJ	Central region (CR), Jiangxi	15	10	A, B1	0.8952±0.0704	0.0068±0.0039
Xinfeng	XF	Central region (CR), Jiangxi	5	2	A, B1	0.4000±0.2373	0.0149±0.0095
Xinyang	XY	Central region (CR), Henan	29	12	A, B1, B2	0.6626±0.1010	0.0086±0.0046
Hanzhong	HZ	Central region (CR), Shaanxi	27	12	A, B1, B2	0.8006±0.0763	0.0144±0.0075
Guizhou	GZ	Southwestern region(SW), Guizhou	29	16	A, B1, B2	0.8966±0.0469	0.0191±0.0097
Dechang	DC	Southwestern region (SW), Sichuan	12	9	A, B2	0.9545±0.0467	0.0148±0.0081
Fuling	FL	Southwestern region (SW), Chongqing	27	14	A, B2	0.8803±0.0527	0.0194±0.0099
Guangxi	GX	Southwestern region (SW), Guangxi	13	9	A, B1, B2	0.9231±0.0572	0.0223±0.0118
Fuzhong	FZ	Southwestern region (SW), Guangxi	5	5	A, B1	1.0000±0.1265	0.0159±0.0100
Xilin	XN	Southwestern region (SW),Guangxi	20	12	A, B1, B2	0.8737±0.0653	0.0188±0.0098
Diandongnan	DDN	Southwestern region (SW), Yunnan	29	4	A, B2	0.9000±0.1610	0.0168±0.0106
Yanjin	YNJ	Southwestern region (SW), Yunnan	5	5	A, B2	1.0000±0.1265	0.0206±0.0129
Dehong	DH	Southwestern region (SW), Yunnan	31	17	A, B1, B2	0.8796±0.0481	0.0132±0.0068

Note: n = sample size; k = number of haplotypes, all 22 populations of China were grouped into three regional groups.

### Population Structure

To further determine the location of the probable center for domestication of the two swamp buffalo mtDNA lineages among the three main hypothetical regions of swamp buffalo domestication, we performed detailed comparative analyses of the diversity for each lineage. We separately performed several AMOVA analyses at different hierarchical levels (Table 2),and analyzed nucleotide and haplotype diversity within lineage A and B for the three defined regional groups (Table 3), including Southeastern (DL, HA, SQ, XL, WZ, FA), Central (HZ, XY, JH, BH, ES, XJ, XF) and Southwestern China (DN, XN, GX, GZ, DC, YJ, DH, FZ, FL). F_ST_ values were calculated for all the possible population pairs. In lineage A, the Matrix of significant F_ST_ P values showed significant differences between Southwestern and Southeastern (P<0.01), Southwestern and Central (P<0.01)and no significant differences existed between Southeastern and Central (P>0.05) (Table 4). Notably, increased haplotype and nucleotide diversity was observed in lineage A of swamp buffaloes in Southwestern China (Table 3). The nucleotide diversity of the Southwestern group (0.0033±0.0019) is higher than those in the remaining two regional groups (Southeastern: 0.0022±0.0014, Central: 0.0017±0.0013) (Table 3). These analyses allowed us to hypothesize Southwestern China as the most likely location for the domestication of lineage A, and hence, a strong candidate for the primary center of swamp buffalo domestication. Indeed, lineage A is by far the most frequent and was observed in all populations in this study (Table 1).

**Table 2:** Analyses at different hierarchical levels between Southwestern group and remaining groups.

Grouping	Within populations (%)	Among populations withingroups (%)	Among groups (%)	F_ST_
No grouping in Chineseswamp buffalo	99.43	0.57 (P = 0.35)		0.006(P = 0.29)
Southwestern versus remaining	99.16 (P = 0.35)	−0.06 (P = 0.36)	0.90 (P = 0.09)	
Southwestern (no DH)versus remaining	98.91 (P = 0.37)	−0.23 (P = 0.33)	1.32 (P = 0.05)	
Southwestern (+FA) versus remaining	98.65 (P = 0.37)	−0.60 (P = 0.35)	1.95 (P = 0.02)	

**Table 3:** Diversity comparison among the three hypothesized domestication centers in China.

Lineage	Region	N	K (k)	Haplotypediversity(SE)	Nucleotidediversity(SE)	Fu’s F_S_ test	Tajima’s D test
**Lineage A**	Southeastern	106	38(29)	0.9518±0.0101	0.0022±0.0014	−27.528 (P = 0.00)	−2.353(P = 0.001)
	Central	117	43(31)	0.9565±0.0096	0.0017±0.0013	−28.250 (P = 0.00)	−2.296(P = 0.00)
	Southwestern	129	49(38)	0.9721±0.0059	0.0033±0.0019	−26.516 (P = 0.00)	−2.278(P = 0.00)
**Lineage B**	Southeastern	34	20(16)	0.9768±0.0167	0.0071±0.0038	−17.772 (P = 0.00)	0.248(P = 0.66)
	Central	27	14(11)	0.9601±0.0259	0.0066±0.0036	−9.717 (P = 0.001)	0.865(P = 0.83)
	Southwestern	42	22(17)	0.9826±0.0093	0.0069±0.0037	−21.166 (P = 0.00)	−0.867(P = 0.21)

Note: N = sample size, K (k) = the number of haplotypes (unique haplotype)**.**

**Table 4:** Population pairwise F_ST_, matrix of significant Fst P values.

Lineage	Region	Southeastern	Central	Southwestern
**Lineage A**	Southeastern	0.000		
	Central	−0.001	0.000	
	Southwestern	0.035**	0.042**	0.000
**Lineage B**	Southeastern	0.000		
	Central	0.003	0.000	
	Southwestern	0.100**	0.023	0.000

Note: **stand for extremely significant level = 0.01.

However, a more complex pattern of diversity was detected in lineage B using the similar analysis (Table 3 and Table 4), which prevent the unambiguous pinpointing of a single region for the domestication of the lineage B.

### Population Expansions

**Figure 5:**
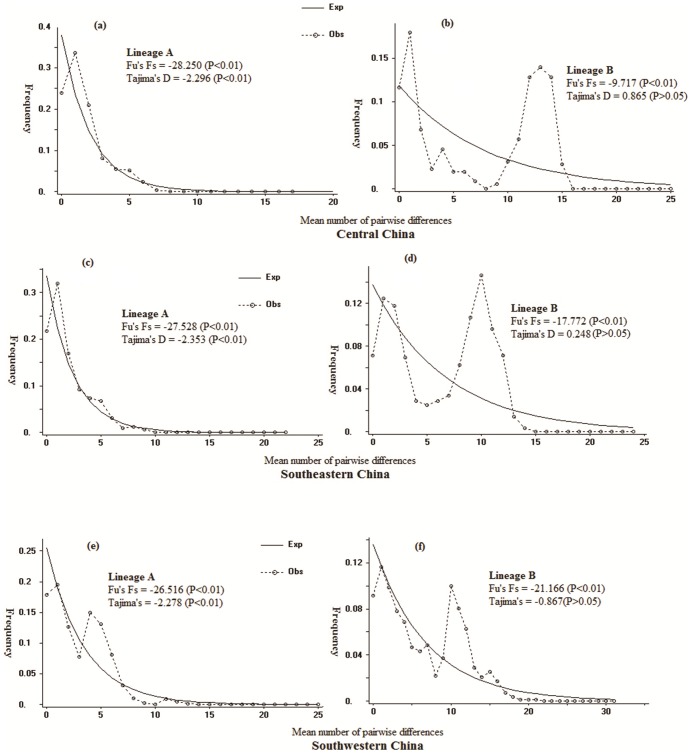
Mismatch distribution and test of neutrality for three hypothesized domestication centers of Chinese buffalo (Southeastern, Central and Southwestern China).

The star-like phylogeny of lineages A and B (sublineages B1 and B2) in the network (Fig. 4) is a sign of population expansions. The sign of a population expansion of lineages A and B was also demonstrated by the bell-shaped curve of the mismatch pairwise distributions (Fig. 5). There was a large negative Fu’s Fs value (−21.80, P<0.005) for the complete dataset of Chinese swamp buffaloes, showing that there were two major peaks at around 1 and 32. These results suggested at least two expansion events in the demographic history of Chinese swamp buffalo populations. Lineages A and B yielded significantly large negative Fu’s Fs test values of −26.32 (P<0.005) and −24.73 (P<0.005), respectively. For sublineages B1 and B2, the Fu’s Fs values are −21.09 (P<0.005) and −13.27 (P<0.005), respectively (Table S3). These analyses uniformly indicated that lineages A and B had undergone three population expansion events.

### Phylogenetic Analysis of Chinese Buffaloes and Swamp Buffalos Outside China

**Figure 6:**
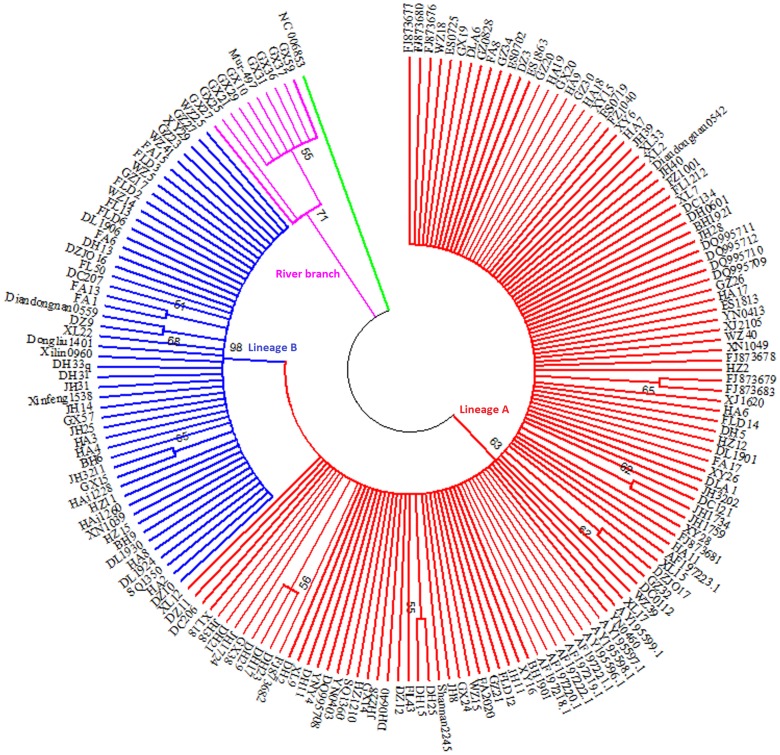
ML tree of Chinese buffalo and Brazil, Italy, Philippine and Thailand buffalo. All the buffalo haplotype outside China are named with NCBI accession number.

In order to investigate the relationship of swamp buffaloes between within and outside China, we compared our data with published mtDNA Control Region sequences of swamp buffaloes, raised in Thailand, Philippines, Brazil and Italy. For a complete alignment we then truncated our sequences to 495 bp. ML tree revealed that all the buffalo sequences outside China are incorporated in lineage A, no sequences fell into lineage B (Fig. 6).

## Discussion

### Genetic Evidence

Previous studies observed that genetic diversity of modern population generally decreases with increasing distance from the domesticated center [33], [34]. In our present study, the genetic diversity comparison of Southwestern Chinese buffalos (DN, XN, GX, GZ, DC, YJ, DH, FZ and FL) with other hypothesized domestication centers (Southeastern and Central China) indicated that Southwestern China may be the most likely location for the domestication of the lineage A, and, thus, a strong candidate for the primary center of swamp buffalo domestication.

As the animals from domestication center can comprise the backbone of the network structure, show lower model value of pairwise difference, and harbor larger amount of unique haplotypes, Chen and his colleagues [35] use these three genetic indexes to support Indus valley as the primary domestication center of domesticated zebu. In this study, the same approaches were used to figure out where is the potential domestication center of swamp buffalo. First, the Southwestern populations revealed a complete backbone structure for the network of the lineage A (Fig. 1b and Fig. 3); second, the modal value [35] of the mtDNA mismatch distribution for the Southwestern China region is higher than the modal value generated for the lineage A of the Central and Southeastern China regions, suggesting that the expansion of the lineage A in Southwestern China region predates that within the Central and Southeastern China regions (Fig. 5); last but not least, the Southwestern China region displays a larger number of unique haplotypes for this lineage A compared with both the Central and Southeastern China regions (Table 3).

It is also worth noticing that three populations (FL, GX, and YJN) within six swamp buffalo populations (BH, FA, GZ, FL, GX, and YJN) with extremely high nucleotide diversity (>0.0190) among 22 Chinese swamp buffalo populations belonged to Southwestern group (Table 1). In fact, though BH and FA belonged to Central and Southeastern group respectively, BH population is native to Northern Hunan province which is adjacent to Guangxi region [18] (Fig. 1). Therefore, it is reasonable to propose that Southwestern region in China appears to be the most likely area where the first domestic Chinese swamp buffalo appeared. To further prove the assumption, all 22 swamp buffalo populations were grouped into two geographical regions (Southwestern versus Central+Southeastern), 0.90% (P = 0.09) of variation was among groups. In the previous microsatellite makers study, DH population revealed very high genetic diversity. Therefore, we do not think the DH samples observed in this study can stand for really pure DH swamp buffalo population [24]. So, the nucleotide diversity value in the DH population was definitely underestimated. If the DH population was removed from the Southwestern group, two geographical regions (Southwestern versus Central+Southeastern), 1.32% (P = 0.05) of variation was among groups. The new result also supported that the Southwestern region in China seems to be the most likely origin site of the first Chinese domestic swamp buffaloes. As expected, two geographical regions (Southwestern+FA population versus Central+Southeastern), 1.95% (P = 0.02) of variation was among groups (Table 2). The result further supported that the Southwestern China is most likely to have first witnessed the domestication of Chinese swamp buffaloes.

However, when similar analyses were performed for the lineage B, a more complex pattern of diversity was detected, whereby some haplotypes derived from the presumed ancestral lineage B haplotypes were observed at high frequencies, whereas other derivative haplotypes were observed only in specific locales. The most notable case is the lineage B haplotype observed to have two sublineages, B1 and B2 in the network, were not supported by ML and Bayesian tree for the low bootstrap value (Fig. 2 and 3). Although phylogenetic trees produced on the basis of sequenced genes or genomic data can infer evolutionary insight, they have important limitations. They do not necessarily accurately represent the species evolutionary history [36]. Phylogenetic trees also have trouble depicting micro-evolutionary events. Phylogenetic networks are used when bifurcating trees are not suitable, due to these complications which suggest a more reticulate evolutionary history of the organisms sampled [37]. In addition, our population expansion analysis indicated that lineage B had undergone two population expansion events. For the above case, we are inclined to believe that the lineage B has two sublineages, B1 and B2, shown up in the network.

Our phylogeographic analysis revealed a weak geographical structure of mtDNA variation in Chinese swamp buffalo types. This phenomenon was also detected in goats including Chinese domestic goats [38]–[40], Chinese sheep [41], Chinese donkeys [42] and Chinese yaks [43]. A possible explanation is that there was strong gene flow among Chinese swamp buffalo populations along the Yangtze River valley and adjacent rivers throughout history. As the economy develops and modernizes in China, swamp buffaloes are seldom herded in the developed regions in Southern China. So our samples were collected from remote mountainous villages of geographical distribution locations. We do not think that the very recent transportation of buffaloes among different regions in Southern China leads to any significantly geographical structure in Chinese swamp buffalo populations. It would be reasonable to conclude that frequent trade and transportation of buffaloes along the Yangtze valley happened throughout history. The fact that Chinese scientists classified all Chinese swamp buffaloes as one breed based on the similarity of body size, outward appearance, coat and biological characteristics is appropriate. Chromosome karyotype [18] also supports the weak geographical structure in Chinese swamp buffalo populations.

**Figure 7:**
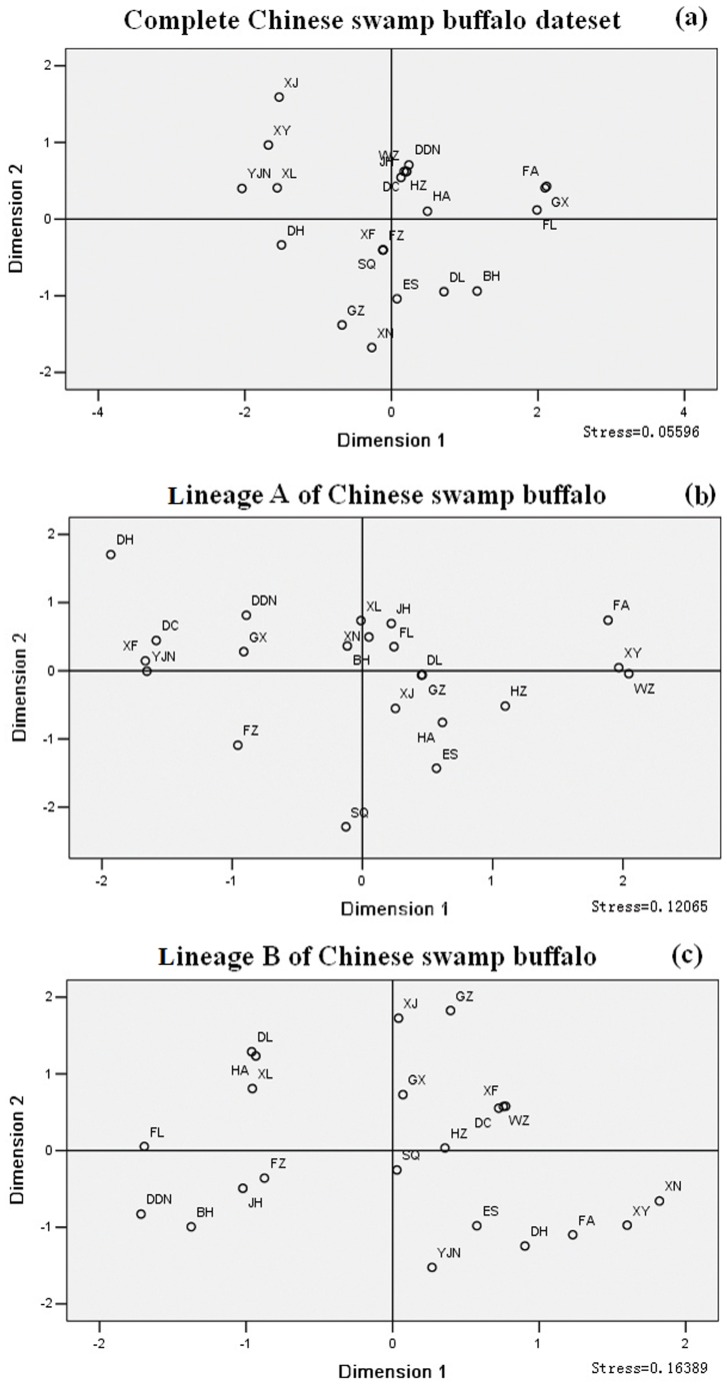
Multidimensional scaling plot of pairwise F_ST_ values between 22 Chinese swamp buffalo populations.

The genetic history of the modern Chinese swamp buffalo was likely to have been associated with inter-regional cultural interactions and population movements in the past. According to the historical records, several immigration upsurges of the human population occurred throughout over 3000 years of Chinese history due to warfare [44]. The immigration movements of human population especially in Southern China caused a parallel flow and merging of swamp buffaloes. It is reasonable that people in Southern China would prefer to take along buffaloes during their migratory movements, as buffaloes have the ability to survive in the similarly humid climate of Southern China and contribute immensely to the agricultural economy through milk, meat, hides and draught power. Buffaloes have become an indispensable part of the property, possession and agricultural tool of rural farmers for centuries [2]. In addition, frequent trade of buffaloes which play a crucial role in rice cultivation in the Southern China further improved the homogeneity of swamp buffaloes in China. Then, all these events contributed greatly to the formation and development of a weak phylogeographical structure of swamp buffaloes. However, even though extensive gene flow existed, we also detected the weak differentiation among 22 Chinese swamp buffalo populations (Fig. 7). Our result was consistent with that based on microsatellite markers [24].

### Archaeological and Ethnographic Evidence

The wild ancestor of *B. bubalis* was *B. arnee*. The present distribution of *B. arnee* is in northeastern India, Nepal, Thailand and Sri Lanka [45], [46], but its distribution in prehistory was much broader, stretching from northwestern South Asia to Mainland Southeastern Asia, based on archaeological remains [47], [48]. Zooarchaeological studies indicate that *B. bubalis* was first domesticated in northwestern part of South Asia before the second millennium BC [48], [49]. These buffalo were likely the river type, as suggested by the modern DNA study [11], [12].

A DNA study of modern swamp buffalo from Southeastern Asia suggests that Thailand appears to have been the center of domestication for this species [15]. Nonetheless, the previous study [15] analyzed shorter Control Region (158 bp) of the much smaller sample size (80 animals) than our current study, which may lose useful genetic information. Furthermore, this study did not incorporate swamp buffaloes outside Southeast Asia in phylogenetic analysis. However, our ML tree construction of Chinese buffaloes and Southeastern Asian buffaloes mt DNA sequences revealed that all the buffalo sequences outside China are incorporated in lineage A, no sequences fell into lineage B. That means China has higher probability as the domestication center of swamp buffaloes; however, more buffalo samples from Southeastern Asia should be collected to confirm this conclusion. The earliest dates for the appearances of domestic swamp buffalo remains in Southeastern Asia fall into the mid first millennium BC, exemplified by Ban Tamyae in central Thailand (600–200 BC) [50], Ban Chiang in north Thailand (around 300 BC) [51], and Phum Snay in northwestern Cambodia (200 BC–AD 240) [52]. This animal appears to have been used for ploughing [51], burial offering, and consumed as food [52].

In China, no *B. arnee* has been found, and no securely identified *B. bubalis* remains dating to the Neolithic or Bronze Age have been reported in archaeological record [19]. The buffalo remains uncovered from Neolithic contexts belong to *B. mephistopheles* whenever identifiable to the species. When *B. bubalis* was mentioned in the archaeological reports, it was normally used as a general term for *Bubalus sp.*

If swamp buffalo was first domesticated in Southwestern China, as suggested by the DNA data discussed above, the most crucial areas for finding the archaeological evidence are Yunnan and Guanxi provinces. There are five prehistoric sites in these regions reporting the discovery of buffalo remains (Table S4). The buffalo from Tangzigou in Baoshan, Yunnan (ca. 6000 BP) was identified as *B. mephistopheles*, based on the morphology of a skull [53]. The *B. bubalis* remains were reported as being unearthed at Haimenkou in Jianchuan, northwest Yunnan, dating to the late part of the first millennium BC [54]. However, the dates are controversial and the deposits of the site may have been disturbed [55]. Therefore, the data from Haimenkou are not reliable. Buffalo bones from three other sites were assigned to *Bubalus* sp. mainly because the absence of horncorns in the faunal assemblages which otherwise would help us identify buffalo species. The lack of well identified buffalo remains from Southwestern China prevents us from using current zooarchaeological data to either support or reject the hypothesis regarding the domestication of buffalo based on DNA research.

Furthermore, there are plenty of records from ancient texts and art presentations for the first appearances of domestic buffalo in Southwestern China. As recorded in “Nanman Xinanyi zhuan” in *Hou Han Shu*, during the Eastern Han period (AD 25–220), Yongchangjun (Yunnan region today) “paid elephant ivory, water buffalo and zebu as tribute” to the Han court. This indicates that Yunnan was known, at latest, by the 1^st^ century AD as a region which produced domestic buffalo. Bovid images, some identifiable as wild and domestic buffalo, were depicted on rock paintings in Cangyuan, Southwestern Yunnan, dating to AD 1^st^–6^th^ Centuries. Sculptures of domestic buffalo, depicted as drought and mounted animal, are seen on bronze drums (AD 3^rd^ –6^th^ Century) from Guangxi. Clay models of buffalo-plough-rice paddy complex have been found in Guangdong, dated to the Western Jin dynasty (AD 265–316) [20]. These data suggest that domestic buffalo appeared in Yunnan probably by the first century AD and gradually spread to other parts of southern China. More archaeological research is needed to understand whether or not this animal went through a process of domestication before the 1^st^ century AD in Southwestern China.

Buffalo sacrifice has been reported as an important ritual practice among several ethnic groups in Southwestern China, particularly in southern part of Yunnan. In the Wa people, for example, buffalo sacrifice is conducted for major ritual events and festivals [56]. It is notable that the ancient rock art with buffalo images was found in the same area where the Wa people live today. Similar buffalo sacrifice ritual has been recorded from many areas in Southeastern Asia [57], suggesting a close cultural tradition associated with buffalo in the region including Southwestern China.

Cultural interactions between Southwestern China and Southeastern Asia existed in ancient times, through many trade networks [58], including the Southwestern Silk Road connecting Sichuan via Yunnan and Burma with South Asia [59]. The material items transported through these routes include precious stones, salt, animals and tea [59]. These inter-regional interactions, likely also including migrations of human populations, may have facilitated the movement of water buffalo between Southeastern Asia and Southwestern China. It is unclear, however, which directions that buffalo was moved in this broad region. Based on the current archaeological information we cannot exclude the possibility that buffalo was first domesticated in Southwestern China and spread to other areas.

### Conclusion

Previous studies have shown that China may have been the domestication center of swamp buffalo [11], [12], [23], [25]. This survey examined the mtDNA Control Region diversity of 455 individuals from 22 Chinese swamp buffalo populations and 16 river buffaloes from Chinese Guangxi population. Phylogenetic analyses revealed that there were two previously defined lineages A and B in Chinese swamp buffalo populations, showing multiple maternal origins of swamp buffalo. Based on genetic evidence, the results of our current study support that Southwestern China is most likely to be the center where Chinese domestic swamp buffaloes first appeared. Since there is no evidence in the archaeological record suggesting that the process of buffalo domestication took place in China, and domestic buffalo occurred in Southeastern Asia (by the 2^nd^ century BC) earlier than it did in China (by the 1^st^ century AD), it is still premature to claim that Southwestern China was the original center of buffalo domestication. There are three possible scenarios: (1) swamp buffalo was domesticated in multiple centers, including Southeastern Asian and Southwestern China; (2) swamp buffalo was first domesticated in Southeastern Asia, and then introduced to Southwestern China before spreading to the adjacent regions; and (3) swamp buffalo was first domesticated in Southwestern China, but archaeologists have not discovered the early buffalo remains. More DNA tests on buffalo from Southeastern Asia, particularly Myanmar, Laos, Vietnam, and Thailand, need to be done in order to compare with the data from China. The multidimensional display of population pairwise F_ST_ values and AMOVA analysis showed weak geographical structuring and weak genetic differentiation because of long-term strong gene flow among swamp buffalo populations caused by extensive transportation of buffaloes and frequent human movements along the Yangtze River in history. The lineage of river buffalo detected in Guangxi swamp buffaloes suggested that the imported river buffalo breeds led to the mixture of Chinese local swamp buffaloes.

## Supporting Information

Figure S1 **ML tree of Chinese swamp buffalo without cutting off reliability percentages (RP) below 50%.**
(TIF)

Table S1 **Swamp buffalo D-Loop sequences retrieve from NCBI database.**
(XLSX)

Table S2 **Polymorphic nucleotide sites of 148 swamp buffalo haplotypes.**
(DOC)

Table S3 **Total genetic diversity indexes in Chinese water buffaloes.**
(DOC)

Table S4 **Archaeological sites associated with buffalo remains dating to the Holocene period.**
(DOCX)
