# Supplementary figures and images for: Phylogeography and Domestication of Chinese Swamp Buffalo
Source: PLoS One. 2013 Feb 20;8(2):e56552. doi: 10.1371/journal.pone.0056552 (PMC3577850; doi:10.1371/journal.pone.0056552)

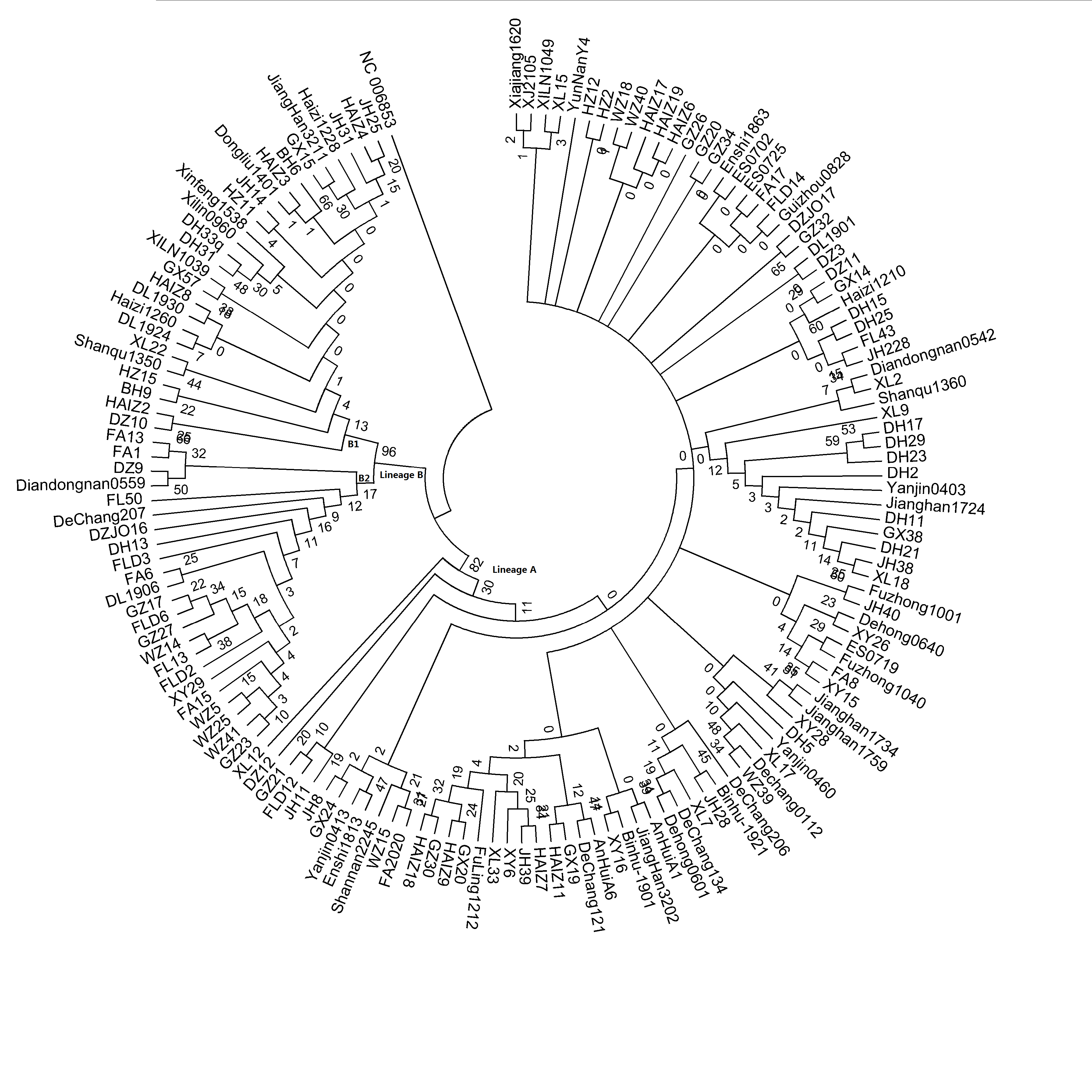

Supplement: Figure S1 — ML tree of Chinese swamp buffalo without cutting off reliability percentages (RP) below 50%. (TIF) [file pone.0056552.s001.tif]
