# Supplementary material for: Phylogeography and Domestication of Chinese Swamp Buffalo
Source: PLoS One. 2013 Feb 20;8(2):e56552. doi: 10.1371/journal.pone.0056552 (PMC3577850; doi:10.1371/journal.pone.0056552)
Supplement: Table S2 — Polymorphic nucleotide sites of 148 swamp buffalo haplotypes. (DOC) [file pone.0056552.s003.doc]

**Table S2** Polymorphic nucleotide sites of 148 swamp buffalo haplotypes

| Position  Haplotype | 11111 1111112222 2222222222 2222222222 3333333333 3333333333 3333334444 4444444444 4555555555 5555566677 7777777778 8999  3367701123 3456671122 3335555677 7888888899 0111122233 3345555578 8899992334 4666667888 9000122234 5677815901 2356666786 7233  0595721603 5413600936 3482678047 8124567836 5345813413 4941347950 2725679372 9234564239 8238248952 0747184155 3813469668 1402 |
| --- | --- |
| DL1 | ACCACCTACT AGAACTCTTG ATTTATAGAA GACCAAACAA ACAAAGTCAT GCCGTCCCCC AACGGTAGTC CGTCTTTCAC GCTCCCCCTC CCCACACGCT AGGTAAATAG CGAC |
| XY16 | .......... .......... .......... .......... .......... .......... ......G... .......... .......... .......... .......... .... |
| DH5 | ....T..... .......... .........G .....G.... .......... .......... ......G... .......... .......... .......... .......... .... |
| ES1 | G......... .......... .........G .......... .......... .......... ......G... .......... .......... .......... .......... .... |
| ES11 | .......... .......... .........G .......... .......... .......... ......G... .......... .......... .......... .........T .... |
| GZ20 | .......... .......... .........G .......... .......... .......... ......G... .......... .......... .........C .......... .... |
| FL1212 | .......... .......... .........G .......... .......... .......... ......G... .......... .......... ....T..... .......... .... |
| HA17 | .......... .......... .........G .......... ...G...... .......... ......G... .......... .......... .......... .......... .... |
| HZ12 | .......... .......... .........G .......... .......... .....T.... ......G... .......... .......... .......... .......... .... |
| ES9 | .......... .......... .........G .......... .......... .......... ....A.G... .......... .......... .......... .......... .... |
| FZ1040 | .......... .......... .........G .......... .......... .......... ....A.G... .......... .......... T......... .......... .... |
| FZ1001 | .......... .........A .........G .......... .......... .......... ......G... .......... .......... .......... .......... .... |
| JH40 | .......... .........A .........G .......... .......... .......... ......G... .......... .......... T......... .......... .... |
| FLD14 | .......... .......... .........G .........G .......... .......... ......G... .......... .......... .......... .......... .... |
| XY28 | .......... .......... .........G ....T..... .......... .......... ......G... .......... .......... .......... .......... .... |
| FLD12 | .......... .......... .........G .......... .......... A......... ......G... .......... .......... .......... .......... .... |
| GZ21 | .......... .......... .........G .......... .......... A......... ......G... .......... .......... .......... .A........ .... |
| GZ0828 | .......... .......... .........G .......... .......... .T........ ......G... .......... .......... .......... .......... .... |
| HA7 | .......... .......... .........G .......... .......... .T........ ......G... .......... ....T..... .......... .......... .... |
| JH39 | .......... .......... .........G .......... .......... .......... ......G... .......... ....T..... .......... .......... .... |
| XY6 | .......... .......... .......... .......... .......... .......... ......G... .......... ...TT..... .......... .......... .... |
| WZ18 | .......... .......... .........G .......... .......... .......... ......G... .......... .......... .......... C......... .... |
| DLA6 | .......... .......... .........G .......... .......... .......... ......G... .......... .......... ......T.T. .......... .... |
| DC121 | .......... .......... .........G .......... .......... .......... .......... .......... .......... ..T...T.T. .......... .... |
| GX19 | .......... .......... .........G .......... .......... .......... ......G... .......... .......... ......T... .......... .... |
| XL33 | .......... .......... .........G .......... .......... .......... ......G... .......... ....TT.... ......T... .......... .... |
| HA11 | .......... .......... .........G .C........ .......... ..T....... ......G... .......... .......... ......T... .......... .... |
| GZ30 | .......... .......... .........G .......... .......... .......... ......G... .......... .T..T..... ....T.T... .......... .... |
| HA18 | .......... .......... .........G .......... .......T.. .......... ......G..T .......... .T........ ....T..... .......... .... |
| GX20 | .........C .......... .........G .......... .......... .......... ......G... .......... .T........ .......... .......... .... |
| HA9 | .......... .......... .........G .......... .......... .......... ......G... .......... .TC....... ......T... .......... .... |
| BH1901 | .......... .......... .......... .......... .......... .......... ......G... .......... .......... .......... .....G.... .... |
| DDN0542 | .......... .......... .........G .......... G......... ....C..... ......G... .......... .......... .......... .....G.... .... |
| GZ34 | .......... .......... .........G .......... .......... .......... ......G... .......... .......... .......... .....G.... .... |
| YJN0460 | .......... .......... .........G ..T....... .......... .T........ ......G... .......... .......... .......... .....G.... .... |
| JH11 | .......... .......... .........G .......... .......... A......... ......G... .......... .......... .......... .....G.... .... |
| JH1759 | .......... .......... .........G ....G..... .......... .......... ......G... .......... .......... .......... .....G.... .... |
| DZJO17 | .......... .......... .........G .......... .......... .T........ .G....G... .......... .......... .......... .......... .... |
| GZ32 | .......... .......... .........G .......... .......... .......... .C....G... T......... .......... .......... .......... .... |
| GZ26 | .......... .......... .........G .......... ......C... .......... ......G... .......... .......... .......A.. .......... .... |
| XN15 | .......... .......... .........G .......... .......... .......... ......G... .......... .......T.. .......... .......... .... |
| XL15 | .......... .......... .........G .......... .......... .......... T.....G... .......... .......... .......... .......... .... |
| ES1863 | .....T.... .......... .........G .......... .......... .......... ......G... .......... .......... .......... .......... .... |
| JH3202 | .......... .......... .........G .......... .......... .......... .......... .......... .......... .......... .......... .... |
| DL1901 | .......... ......T... .........G .......... .......... .......... ......G... .......... .......... .......... .......... .... |
| ES1813 | .......... .......... ...C.....G .......... .......... .......... ......G... .......... .......... .......... .......... .... |
| XJ1620 | .......... .A........ .........G .......... .......... .......... ......G... .......... .......... .......... .......... .... |
| HA6 | .......... .......... .........G .......... .......... .......... ......G... .........T .......... .......... .......... .... |
| HZ2 | .......... .......... .........G .......... .......... .......... ...A..G... .......... .......... .......... .......... .... |
| XJ3 | .......... .......... G........G .......... .......... .......... ......G... .......... .......... .......... .......... .... |
| HA19 | .......... .......... .........G .......... .......... .......... ......G... .......... .......... .......... .......... .... |
| JH1734 | .......... .......... .........G ....G..... .......... .......... ......G... .......... .......... .......... .......... .... |
| DZ12 | .......... .......... .........G .......... .......... A.....TT.. ......G... .......... .......... .......... .......... .... |
| FL43 | .......... .......... .........G .......... .......... .......T.. ......G... .......... .......... .......... .......... .... |
| FA2020 | .......... .......... .........G .......... .......... .......... ......G... .......T.. .......... .......... .......... .... |
| WZ15 | .......... .......... .........G .......T.. .......... A......... ......G... .......T.. .......... .......... .......... .... |
| HZ2245 | .......... .......... .........G .......... .......... .......T.. ......G... .......T.. ......T... .......... .......... .... |
| GX24 | .......... .......... .........G .......... .......... .......... ......G... .......... ......T... .......... .......... .... |
| JH8 | .......... .......... .........G .......... .......... A......... ......G... .......... ......T... .......... .......... .... |
| YJN0413 | .......... .......... ...C.....G .......... .......... .......... ......G... .A........ ......T... .......... .......... .... |
| WZ39 | .......... .......... ..C......G .......... .........C .......... ......G... .......... .......... .......... .......... .... |
| XL17 | .......... .......... .........G ..T....... .........C .......... ......G... .......... .......... .......... .......... .... |
| DC0112 | .......... .......... .........G .......... .........C .......... ......G... .......... .......... .......... .......... .... |
| FA8 | .......... .......... .........G .......... .......... .......... ......G... .......... .......... .......... ...C...... .... |
| XY15 | .......... .......... .........G .......... .......... .......... ....A.G... .......... .......... .......... ...C...... .... |
| WZ40 | .......... .......... .........G ........G. .......... .......... ......G... .......... .......... .......... .......... .... |
| XY26 | .......... .......... .........G .......... .......... .......... ......G... ..C....... .......... .......... .......... .... |
| FA17 | .......... .......... .......A.G .......... .......... .......... ......G... .......... .......... .......... .......... .... |
| DC134 | .......... .......... .........G ...T...... .......... .........G ......G... ...T...... .......... .......... .......... .A.. |
| XL7 | .......... .......... .........G ...T...... .......... .......... ......G... .......... .......... .......... .....G.... .... |
| DH0601 | .......... .......... .........G ...T...... .......... .......... ......G... .......... .......... .......... .......... .... |
| BH1921 | .......... .......... .........G ...T...... .......... ........T. ......G... .......... .......... .......... .......... .... |
| JH28 | .......... .......... .........G .......... .......... ........T. ......G... .......... .......... .......... .......... .... |
| DZ3 | .......... .......... .........G .......... .......... .......... ......G... .......... .......... .......... ......G... .... |
| XL2 | .......... .......... .........G .......... G......... .......... ......G... .......... .......... .......... .......... .... |
| DH15 | .......... .......... .C.......G .......... .......... .......T.. ......G... .......... .......... .......... .......... .... |
| DH25 | .......... .......... .C.......G .......... .......... .......... ......G... .......... .......... .......... .......... .... |
| DH0640 | .......... ...G...... ........G. .......... .......... .T.A...T.. ......G... ..C..C..G. ........C. .......... ...C...... .... |
| SQ1360 | .......... .......... .......... .......... G......... .......T.. ......G... .......... .......... .......... .......... .... |
| JH228 | .......... .......... .........G .......... .......... .......T.. ......G... .......... .......... ..T....... .......... .... |
| DH11 | .......... .......... .......... .......... G......... .......T.. ......G... .......... .......... ..T....... .......... .... |
| YJN0403 | .......... .......... .......... .......... G......... .......TT. ......G... .......... .......... ..T....... .......... .... |
| DH21 | .......... .......... .....C.... .......... G......... .......T.. ......G... .......... .......... ..T....... .......... .... |
| GX38 | .......... ........C. .......... .......... G......... .......T.. ......G... .......... .......... ..T....... .......... .... |
| XL9 | .......... .......... .......... .......... G......... .......T.. ......G... .......... ......T... ..T....... .......... .... |
| DH17 | .......... .......C.. .......... .......... G........C .......T.. ......G... .......... .......... ..T....... .......... .... |
| DH23 | .......... .......... .......... .......... G........C ....C..T.. ......G... .......... .......... ..T....... .......... .... |
| DH29 | .......... .......... .......... .......... G........C .......T.. ......G... .......... .......... ..T....... .......... .... |
| DH2 | .......... .......... .......... ....G..... G......... .......T.. ......G... .......... .......... ..T....... .......... .... |
| JH38 | .......... ..G....... .......... .......... G......... .......T.. ......G... .......... .......... ..T....... .......... .... |
| XL18 | .......... .......... .......... .......... G......... .......T.. ......G... .......... .......... ..TG...... .......... .... |
| JH1724 | .......... .......... .......... .......... G.....C... .......T.. ......G... .......... .......... ..T....... .......... .... |
| YJNY4 | .......... .......... .......... .......... G......... .......T.. ......G... .......... .......... ..T...T... .......... .... |
| DC206 | .......... .......... .........G .......... G......... .......T.G ..G...G..T .......... .......... ..T...T... .......... .... |
| HA1210 | .......... .......... .......... .......... .......... ..T....T.. ......G... .......... .......... ..T....... .......... .... |
| GX14 | .......... .......... .......... .......... .......... .......T.. ......G... .......... .......... ..T....... ..A....... .... |
| DZ11 | .......... .......... .......... ...T...... G......... .......T.. ......G... .......... .......... ..T....... ..A....... .... |
| XL12 | .......... .......... .......... ...T...... G......... A.....TT.. ......G... .......T.. .......... ..T....... .......... .... |
| DH31 | ..T....GT. ...GTCT... .......A.. AGTT..G... .TG.GA.... A.....T.TT ....ACG.C. .A.....T.. A......... .....G.... ..AC...... T.GT |
| DH33q | ..T....GT. ...GTCT... .......A.. AGTT..G... .TG.GA.... A.......TT ....ACG.C. .A.....T.. A......... .....G.... ..AC...... T.GT |
| XF1538 | ..T....GT. ...GTCT... .......A.. AGTT..G... ..G.GA.... A.....T.TT ....ACG.C. .A.....T.. A......... .....G.... ..AC...... T.GT |
| HA1228 | ..T....GT. ...GTCT... .......A.. AGTT..G... ..G.GA.... ......T.TT ....ACGAC. .A.....T.. A......... .....G.... ..AC...... T.GT |
| HZ11 | ..T....GT. ...GTCT... .......A.. AGTT..G... ..G.GA.... ......T.TT ....ACG.C. .A.....T.. A......... ....TG.... ..AC...... T.GT |
| BH6 | ..T....GT. G..GTCT... .......A.. AGTT..G... ..G.GA.... ......T.TT ....ACG.C. .A.....T.. A......... .....G.... ..AC...... T.GT |
| GX15 | ..T....GT. ...GTCT... .......A.. AGTT..GT.. ..G.GA..G. ......T.TT ....ACG.C. .A.....T.. A......... .....G.... ..AC...... T.GT |
| JH3211 | ..T....GT. ...GTCT... .......A.. AGTT..GT.. ..G.GA.... ......T.TT ....ACG.C. .A.....T.. A......... .....G.... ..AC...... T.GT |
| HA8 | ..T....GT. ...GTCT... .......A.. AGTT..G... ..G.GA...C ......TTTT ....ACG.C. .A.....T.. A......... ....TGT... ..AC...... T.GT |
| JH31 | ..T....GT. ...GTCT... .......A.. AGTT..G... ..G.GA.... A.....TTTT ....ACG.C. .A.....T.. A......... .....G.... ..AC....C. T.GT |
| HA4 | ..T....GT. ...GTCT... .......A.. AGTT..G... ..G.GA.... ......T.TT ....ACG.C. .A.....T.. A......... .....G.... ..AC...... T.GT |
| JH25 | ..T....GT. ...GTCT... .......A.. AGTT..G... ..G.GA.... ......T.TT ....ACG.C. .A.....T.. A......... .....G.... ..AC...C.. T.GT |
| GX57 | ..T....GT. ...GTCT... .......A.. AGTT..G... ..G.GA.... ......TTTT ....ACG.C. .AC....T.. A......... .....G.... ..AC...... T.GT |
| XN12 | ..T...CGT. ...GTCT... .......A.. AGTT..G... ..G.GA...C ......T.TT ....ACG.C. .AC....T.. A......... .....G.... ..AC...... T.GT |
| DL1930 | ..T....GT. ...GTCT... ...C...A.. AGTT..G... ..G.GA...C ......TTTT ....ACG.C. .A.....T.. A......... .....G.... ..AC...... T.GT |
| HA1260 | ..T....GT. ...GTCT... .......A.. AGTT..G... ..G.GA...C ......T.TT ....ACG.C. .A.....T.. A......... .....G.... ..AC...... T.GT |
| HZ15 | ..T....GT. ...GTCT.C. .......A.. AGTT..G... ..G.GA...C ....C.T.TT ....ACG.C. .A.....T.. A......... .....G.... ..AC...... T.GT |
| JH14 | ..T....GT. ...GTCT... .......A.. AGTT..G... ..G.GA.... ......TTTT ....ACG.C. .A.....T.. A......... .....G.... ..AC...... T.GT |
| SQ1350 | ..T....GT. ...GTCT... .......A.. AGTT..G... ..G.GA...C ......T.TT ....A.G.C. .A.....T.. A......... .....G.... ..AC...... T.GT |
| XL22 | ..T....GT. G..GTCT... .......A.. AGTT..G... ..G.GA.... ......T.TT ....A.G.C. .A.....T.. A......... .....G.... ..AC...... T.GT |
| DL1401 | ..T....GT. ...GTCT... .......A.. AGTT..G... ..G.GA.... ......T.TT ....ACG... .A.....T.. A......... .....G.... ..AC...... T.GT |
| BH9 | ..T....GT. ...GTCT.C. .......A.. A.TT..G... ..G.GA...C ......T.TT ....ACG.C. .A....GT.. A......... .....G.... ..AC...... T.GT |
| DL1924 | ..T....GT. ...GTCT... .......A.. A.TT..G... ..G.GA...C ......T.TT ....ACG.C. .A.....T.. A......... .....G.... ..AC...... T.GT |
| HAIZ2 | ..T....GT. ...GTCT... .......A.. AGTT..G... ..G.G..... ......T.TT ....ACG.C. .A.....T.. A......... .....G.... ..AC...... T.GT |
| HAIZ3 | ..T....GT. ...GTCT... .......A.. AGTT..G... ..G.GA.... ......T.TT ....ACG.C. .A.....T.. A......... .....G.... ..AC...... T..T |
| XN0960 | ..T....GT. ...GTCT... .......A.. AGTT...... .TG.GA.... ......T.TT ....ACG.C. .A.....T.. A......... .....G.... ..AC...... T.GT |
| DZ10 | ..T....GT. ...GTCT... .......A.. AG.T..G... ..G....... ......T.TT ....ACG.C. .A.....T.. A......... .....G.... ..AC...... T.GT |
| DL2 | ..T....GT. ...GTCT.C. .....C.A.. A..T..G... ..G.GA...C A.....TTTT ....ACG.C. .A.....T.. A......... .....G.... ..A....... T..T |
| FA6 | .GT....GT. ...GTCT.C. .....C.A.. A..T..G... ..G.GA...C A.....T.TT ....ACG.C. .A.....T.. A......... .....G.... ..AC...... T.GT |
| FA15 | ..T....GT. ...GTCT.C. .....C.A.. A..T..G... ..G.GA...C A.....TT.T ....ACG.C. .A.....T.. A......... .....G.... ..A....... T..T |
| GZ23 | ..T....GT. ...GTCT.C. .....C.A.. A..T..G... ..G.GA...C A.....TT.T ....ACG.C. .A.....T.. A.C....... .....G.... ..A....... T..T |
| WZ41 | ..T....GT. ...GTCT.C. .....C.A.. A..T..G... ..G.GA...C A.....TT.T .G..ACG.C. .A.....T.. A......... .....G.... ..A..G.... T..T |
| WZ25 | ..T....GT. G..GTCT.C. .....C.A.. A..T..G... ..G.GA...C A.....TT.T ....ACG.C. .A.....T.. A....T.... .....G.... ..A....... T..T |
| WZ5 | ..T....GT. G..GTCT.C. .....C.A.. A..T..G... ..G.GA...C A.....TT.T ....ACG.C. .A.....T.. A......... .....G.... ..A....... T..T |
| FL13 | ..TG...GT. ...GTCT.C. .....C.A.. A..T..G... ..G.GA...C A.....TT.T ....ACG.C. .A.....T.. A......... ....TG.... ..A....... T..T |
| WZ14 | ..T....GT. ...GTCT.C. .....C.A.. A..T..G... ..G.GA...C A.....TT.T ....ACG.C. .A.....T.. A......... ....TG.... ..A....... T..T |
| FLD6 | ..T....GT. ...GTCT.C. .....C.A.. A..T..G... ..G.GA...C A.....TT.T ....ACG.C. .A.....T.. A......... ....TGT... ..A....... T..T |
| GZ17 | ..T....GT. ...GTCT.C. .....C.A.. A..T..G... ..G.GA...C A.....TT.T ....ACG.C. .A.....T.. A......... ....TGT... ..C.C..... T..T |
| GZ27 | ..T....GT. ...GTCT.C. .....C.A.. A..T..G... ..G.GA...C A.....TT.T ....ACG.C. .A.....T.. A....T...T .G..TGTA.. ..A....... T..T |
| XY29 | ..T....GT. ...GTCT.C. .....C.A.. A..T..G... ..G.GA...C A.....TT.T ....ACG.C. .A.....T.. A......... ..T..G.... ..A....... T..T |
| FLD2 | .GT....GT. ...GTCT.C. .....C.A.. A..T..G... ..G.GA...C A.....TT.T ....ACG.C. .A.....T.. A......... ....T..... ..A....... T..T |
| FLD3 | .GT....GT. ...GTCT.C. .....C.A.. A..T..G... ..G.GA...C A.....TT.. ....ACG.C. .A.....T.. A......... .....G.... ..A....... T..T |
| DH13 | ..T....GT. ...GTCT... .....C.A.. A..T..G... ..G.GA...C A.....TT.T ....ACG.C. .A.....T.. A......... .....G.... ..A....... T..T |
| DZJO16 | ..T....GT. ...GTCT.C. .....C.A.. A..T..G... ..G.GA...C ......TT.T ....ACG.C. .A.....T.. A......... .....G.... ..A....... T..T |
| DDN0559 | ..T....GT. ...GTCT.C. .......A.. A.TT..G... ..G.GA...C ......TT.T ....ACG.C. .AC....T.. A......... .....G.... ..AC...... T..T |
| DZ9 | ..T....GT. ...GTCT.C. .......A.. A.TT..G... ..G.GA...C ......TT.T ....ACG.C. .AC....T.. A......... .....G.... ..A....... T..T |
| FA1 | ..T....GT. ...GTCT.C. .......A.. A.TT..G... ..G.GA...C ......TT.T ....ACG.C. .A..C..T.. A......... ....TGTA.. ..AC...... T..T |
| FA13 | ..T....GT. ...GTCT.C. .......A.. A.TT..G... ..G.GA...C ......TT.T ....ACG.C. .A..C..T.. A......... .....G.A.. ..AC...... T..T |
| FL50 | ..T....GT. ...GTCT.C. ....C.TA.. A..T..G... ..G.GA...C ......TT.T ....ACG.C. .A.....T.. A......... ....TGT... ..A....... T..T |
| DC207 | ..T....GT. ...GTCT.C. .......A.. A..T..G... ..G.GA...C A.....TT.T ....ACG.C. .A.....T.. A......... .....G.... ..A....... T... |
