# Supplementary material for: Phylogeography and Domestication of Chinese Swamp Buffalo
Source: PLoS One. 2013 Feb 20;8(2):e56552. doi: 10.1371/journal.pone.0056552 (PMC3577850; doi:10.1371/journal.pone.0056552)
Supplement: Table S3 — Total genetic diversity indexes in Chinese water buffaloes. (DOC) [file pone.0056552.s004.doc]

**Table S3 Total genetic diversity indexes in Chinese water buffaloes**

| **Lineages** | **N** | **K** | **J** | **Fu's F_S_ value** | **Haplotype diversity**  **H±SD** | **Nucleotide diversity**  **π±SD** |
| --- | --- | --- | --- | --- | --- | --- |
| Swamp type | 455 | 148 | 113 | -21.80 | 0.8601±0.00155 | 0.0158±0.0079 |
| Lineage A | 352 | 98 | 84 | -26.32 | 0.7752±0.0239 | 0.0029±0.0017 |
| Lineage B | 103 | 50 | 56 | -24.73 | 0.8932±0.0253 | 0.0084±0.0043 |
| sublineage B1 | 43 | 27 | 32 | -21.09 | 0.9059±0.0391 | 0.0041±0.0023 |
| sublineage B2 | 60 | 23 | 34 | -13.27 | 0.7311±0.0633 | 0.0032±0.0019 |
| River type | 16 | 9 | 31 |  | 0.9080±0.0479 | 0.0095±0.0052 |
| Total | 471 | 157 |  |  | 0.8670±0.0184 | 0.0209±0.0102 |

Note：N=sample size; K=number of haplotypes; J= polymorphic nucleotide site
