# Supplementary material for: Phylogeography and Domestication of Chinese Swamp Buffalo
Source: PLoS One. 2013 Feb 20;8(2):e56552. doi: 10.1371/journal.pone.0056552 (PMC3577850; doi:10.1371/journal.pone.0056552)
Supplement: Table S4 — Archaeological sites associated with buffalo remains dating to the Holocene period. (DOCX) [file pone.0056552.s005.docx]

Table S4. Archaeological sites associated with buffalo remains dating to the Holocene period

| Region | Site | Archaeological culture | Date (BC) | identification | quantity |
| --- | --- | --- | --- | --- | --- |
| Neolithic period | | | | | |
| Inner Mongolia | Xinglonggou | Xinglongwa | 6000-5500 | Cf. *Bubalus* sp. | unknown |
| Inner Mongolia | Shihushan I | Early Yangshao | 4500-4000 | *B. mephistopheles* | 520 NISP |
| Hebei | Jiangou | Longshan | 2500-2000 | *Bubalus* sp. | unknown |
| Henan | Jiahu | Peiligang | 7000-5800 | Cf*. Bubalus* sp. | 7 nisp |
| Henan | Xishan | Early Yangshao | 4500-2800 | *Bubalus* sp. | 5 MIN |
| Shaanxi | Guantaoyuan | Laoguantai | 5300-5000 | *B. mephistopheles* | 1 MIN |
| Shaanxi | Baijiacun | Laoguantai | 5300-5000 | *B. mephistopheles* | 166 NISP |
| Shaanxi | Kangjia | Late Longshan | 2500-2000 | *B. mephistopheles* | 51 NISP |
| Shandong | Wangyin | Dawenkou | 3950-3225 | *B. bubalis* | unknown |
| Shandong | Zhuangxili | Longshan | 2500-2000 | *Bubalus* sp. | unknown |
| Anhui | Shuangdun | Shuangdun | 5000-4000 | *Bubalus* sp. | unknown |
| Anhui | Yuchisi | Dawenkou & Zaolutai | 2800-2600  2500-2300 | *B. mephistopheles*  *B. mephistopheles* | 1 NISP  2 NISP |
| Jiangsu | Dadunzi | Dawenkou | 3800-3000 | *Bubalus* sp. | unknown |
| Henan | Xiawanggang | Yangshao | 2585 | *Bubalus* sp. | 2 NISP |
| Hubei | Chengbeixi | Chengbeixi | 6500-5000 | *B. mephistopheles* | 1 NISP |
| Hubei | Nanmuyuan | Nanmuyuan | 5400-4800 | *B. mephistopheles* | 3 MIN |
| Hubei | Liulinxi | Chengbeixi-Daxi | 5000-4000 | *B. mephistopheles* | 2 MIN |
| Hubei | Weiganping | Daxi | 4400-2700 | *B. mephistopheles* | 8 MIN |
| Hubei | Xisiping | Early Daxi | 4000 | *Bubalus* sp. | 2 MIN |
| Hubei | Sandun | Xuejiagang | 4000-2500 | *Bubalus* sp. | 7 MIN |
| Hubei | Shazui | Early-mid Daxi | 3500 | *Bubalus* sp. | 2 MIN |
| Chongqing | Yuxi | Mid-late Neolithic | 5000-3000 | *B. mephistopheles* | 17 MIN |
| Chongqing | Zhongba | Late Neolihtic - Qin | 2500-200 | *Bubalus* sp. | unknown |
| Hunan | Hujiawuchang | Lower Zaoshi | 5900-5300 | *Bubalus* sp. | unknown |
| Hunan | Shimen Zaoshi | Lower Zaoshi | 5000 | *Bubalus* sp. | unknown |
| Hunan | Chengtoushan | Daxi | 4000-3000 | *Bubalus* sp. | 3 NISP |
| Jiangsu | Sanxingcun | Majiabang | 6500 | *Bubalus* sp. | unknown |
| Jiangsu | Caoxieshan | Majiabang | 4000 | *Bubalus* sp. | unknown |
| Jiangsu | Xudun | Majiabang | 3500 | *Bubalus* sp. | >13 MIN |
| Jiangsu | Longnan | Liangzhu | 3300 | cf. *Bubalus* sp. | 10 NISP |
| Shanghai | Songze | Majiabang  Songze | 5000-4000  4000-3300 | *Bubalus* sp.  *Bubalus* sp. | unknown |
| Shanghai | Yaojiajuan | Songze-Liangzhu | 3300 | *Bubalus* sp. | unknown |
| Zhejiang | Leqingbaishi | Early Neolithic | 8000 | *Bubalus* sp. | 36 NISP |
| Zhejiang | Kuahuqiao | Kuahuqiao | 6000-5000 | *B. mephistopheles* | 765 NISP |
| Zhejiang | Majiabang | Majiabang | 5000-4000 | *Bubalus* sp. | unknown |
| Zhejiang | Luojiajiao | Majiabang | 5000 | *B. mephistopheles* | 39 MIN |
| Zhejiang | Hemudu | Hemudu | 5000-4000 | *B. mephistopheles* | >91 NISP |
| Guangxi | Zengpiyan | Early Neolithic | 10500-5600 | *Bubalus* sp. | unknown |
| Guangxi | Hengxian Jiangkou | Mid Neolithic | 5000-3000 | *B. bubalis* | unknown |
| Guangxi | Gexinqiao | Early Neolithic | 4000 | *Bubalus* sp. | 119 NISP |
| Yunnan | Baoshan Pupiao | Neolithic | 6000 | *B. mephistopheles* | unknown |
| **Bronze Age** | | | | | |
| Hebei | Taixi | Early Shang | 1600-1300 | *B. mephistopheles* | unknown |
| Henan | Yanshi Shangcheng | Early Shang | 1600-1400 | *Bubalus* sp. | unknown |
| Henan | Huayuanzhuang | Middle Shang | 1300-1250 | *Bubalus* sp. | unknown |
| Henan | Yinxu | Late Shang | 1250-1046 | *B. mephistopheles* | >1000 NISP |
| Shaanxi | Baqiao | Early Shang | 1600-1500 | *B. mephistopheles* | 1 NISP |
| Shaanxi | Fengxi | Proto-Zhou | ?-1046 | *B. mephistopheles* | 1 NISP |
| Shandong | Daxinzhuang | Late Shang | 1250-1046 | *Bubalus* sp. | 1 NISP |
| Hubei | Xianglush | Early Shang | 1600-1400 | *B. mephistopheles* | 1 NISP |
| Hubei | Lujiahe | Lujiahe | 1600-1000 | *Bubalus* sp. | unknown |
| Hubei | Zhouliangyuqiao | Zhouliangyuqiao | 1300-1000 | *B. bubalis* | 7 MIN |
| Shanghai | Maqiao | Maqiao | 1900-1100 | *Bubalus* sp. | I NISP |
| Yunnan | Haimenkou | Zhou | 600 | *B. bubalis* | unknown |

Note: this table was modified and updated from Wang and Zhang 2011: table 1

Wang J and Zhang JZ (2011) Shenghuiniu de jiayang/yesheng shuxing chubu yanjiu (Preliminary study on the domesticated or wild characteristics of *Bubalus mephistopheles*). *Nanfang Wenwu* 3(134-139).
